# Supplementary figures and images for: Adapting to Novel Environments Together: Evolutionary and Ecological Correlates of the Bacterial Microbiome of the World’s Largest Cavefish Diversification (Cyprinidae, Sinocyclocheilus)
Source: Front Microbiol. 2022 Mar 14;13:823254. doi: 10.3389/fmicb.2022.823254 (PMC8964274; doi:10.3389/fmicb.2022.823254)

**A**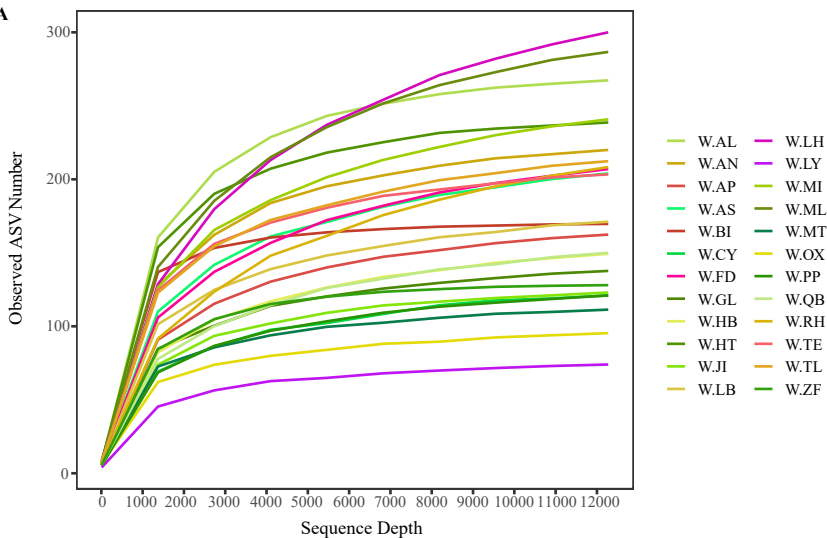**B**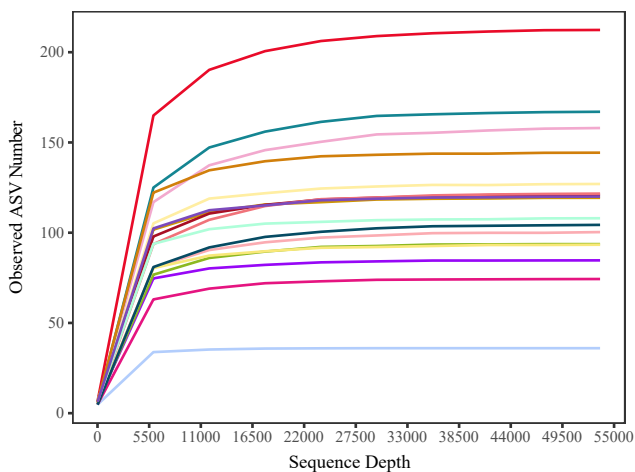**C**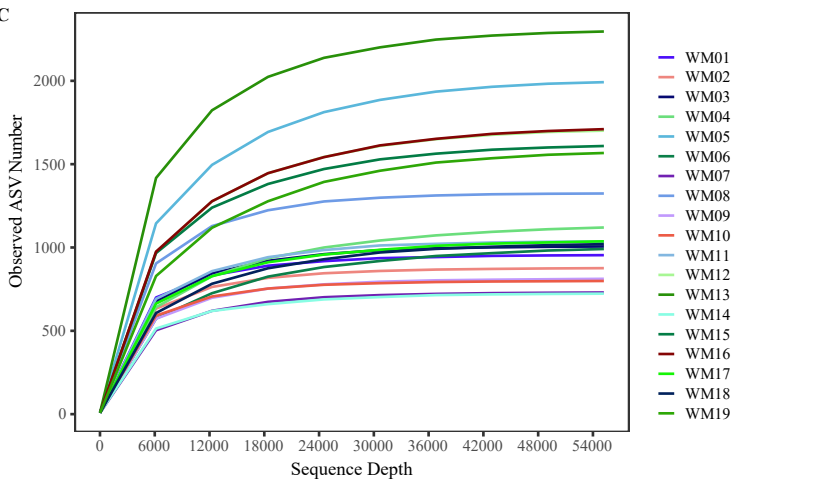

Supplement: Supplementary Figure 1 — Rarefaction curves of each sample. (A) Fecal samples from 24 species of wild Sinocyclocheilus. (B) Fecal samples from 17 species of captive Sinocyclocheilus. (C) Water microbial samples from 19 caves. Different colors of the lines indicate different sample, respectively. [file Data_Sheet_1.PDF]

A

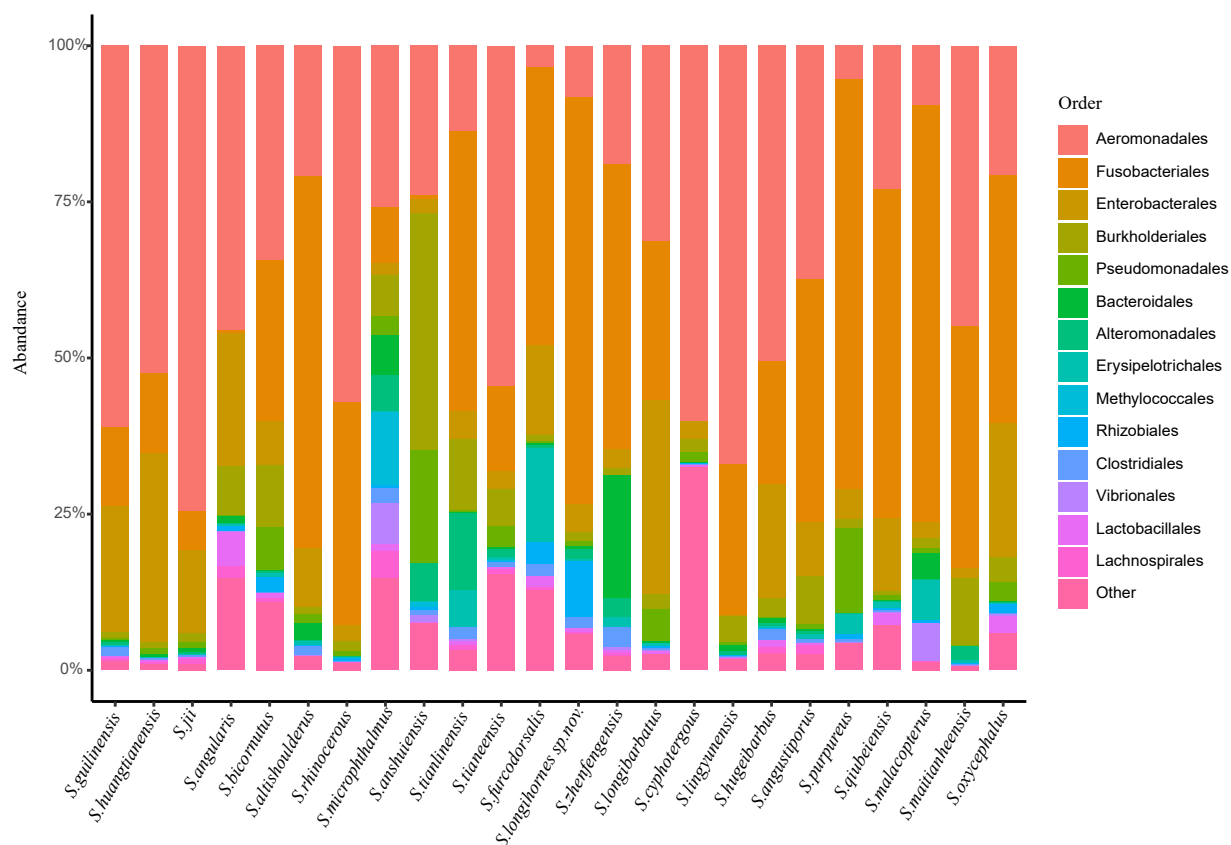

B

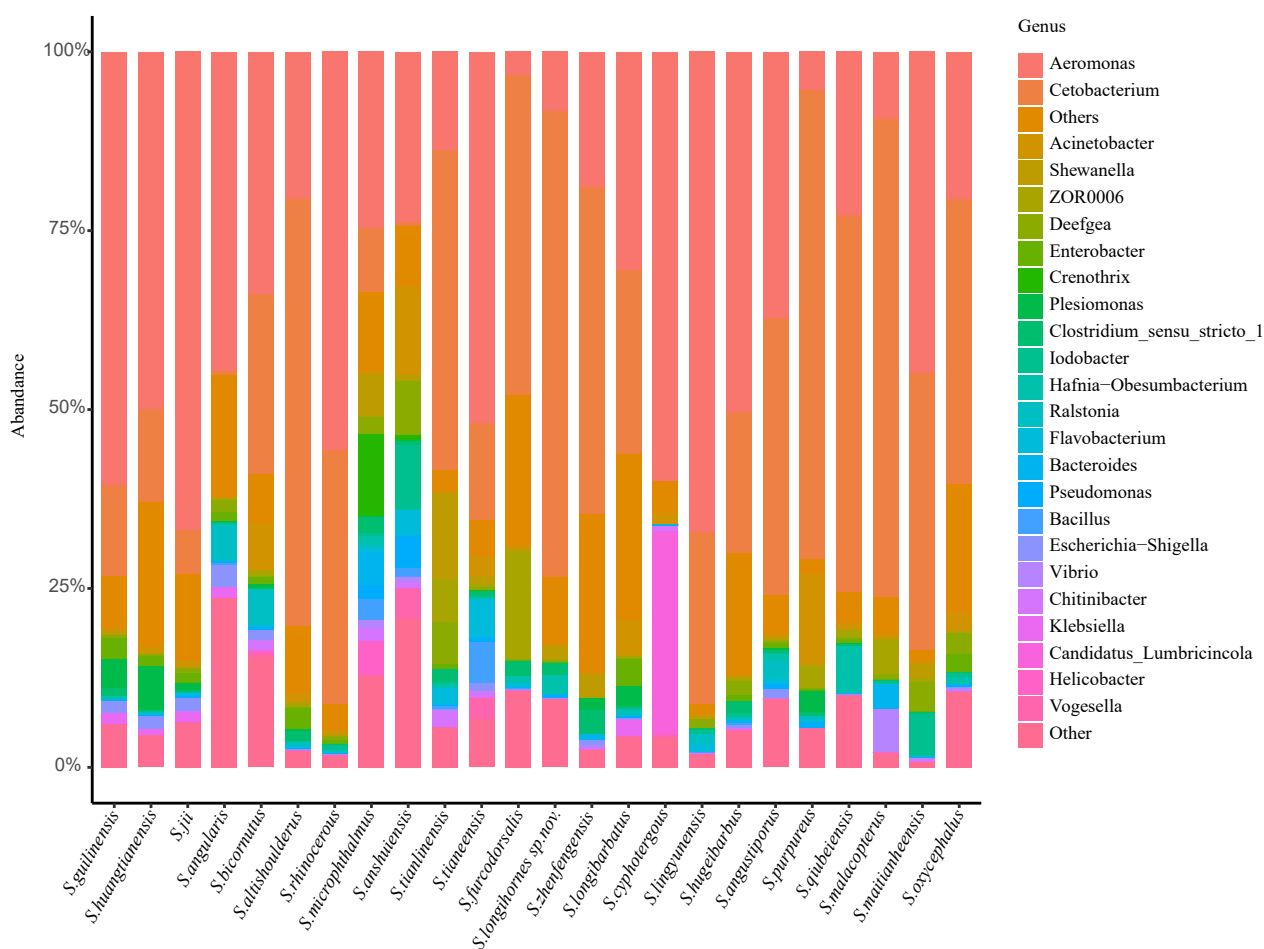

Supplement: Supplementary Figure 2 — Microbial composition of Sinocyclocheilus species. (A) Order level. (B) Genus level. Different colors in the figures indicate the different groups, and details are shown on the right sides of each figure, respectively. [file Data_Sheet_2.PDF]

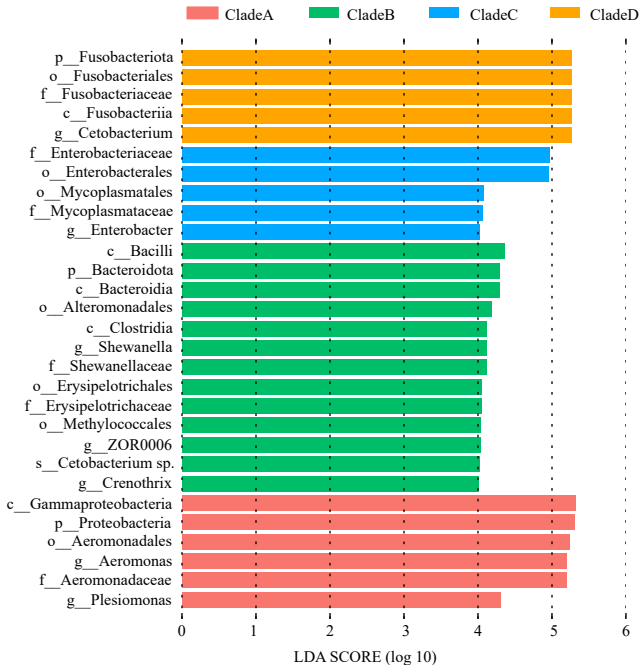

Supplement: Supplementary Figure 3 — LDA Effect Size (LEfSe) method to find out microbiota biomarkers between four Phylogenetic clades. [file Data_Sheet_3.PDF]

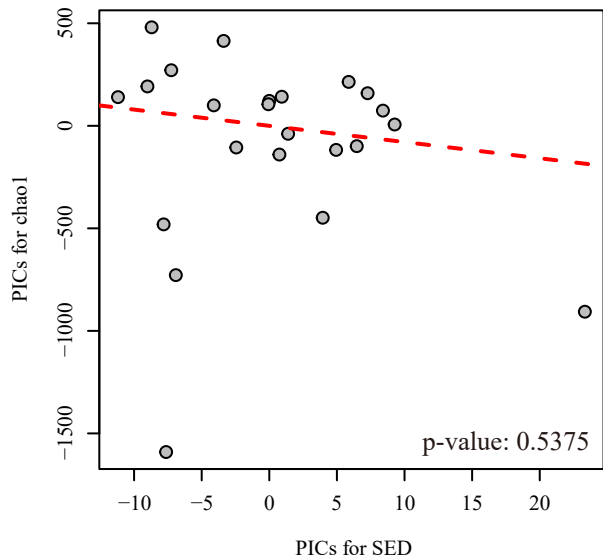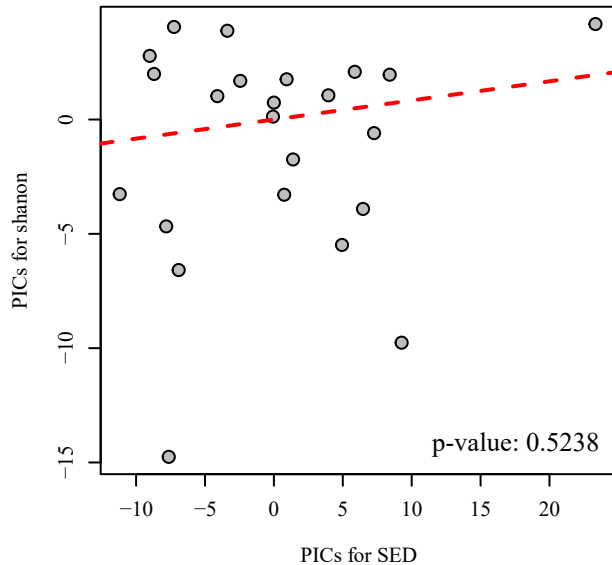

Supplement: Supplementary Figure 4 — The phylogenetic independent contrasts between standard eye diameter (sED) and gut microbial alpha diversity index. [file Data_Sheet_4.PDF]

# bray\_curtis Anosim

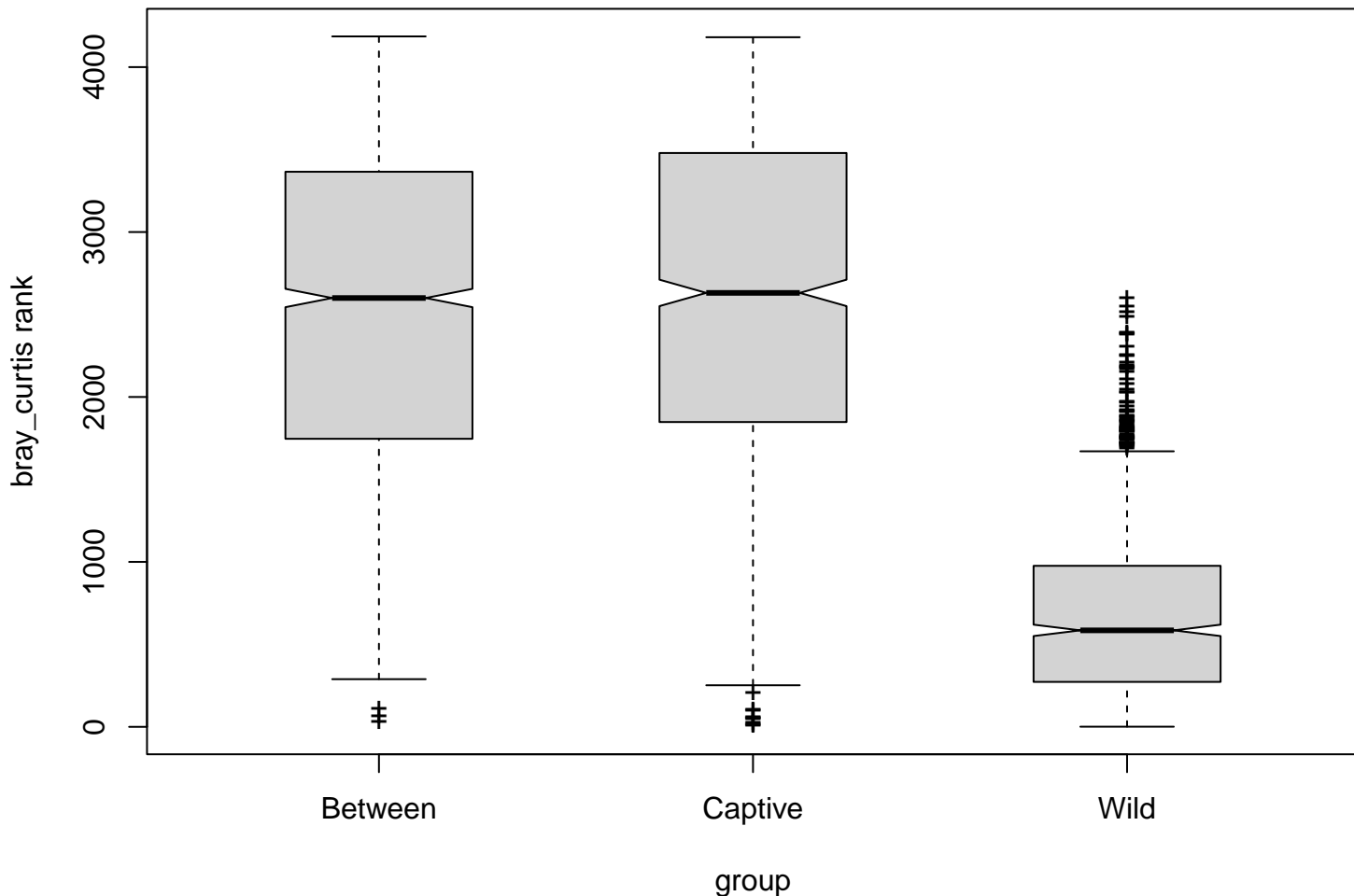

R= 0.428172972429979 p= 0.001

Supplement: Supplementary Figure 5 — Analysis of similarities (Anosim) test based on Bray–Curtis distance for wild group and captive group. [file Data_Sheet_5.PDF]

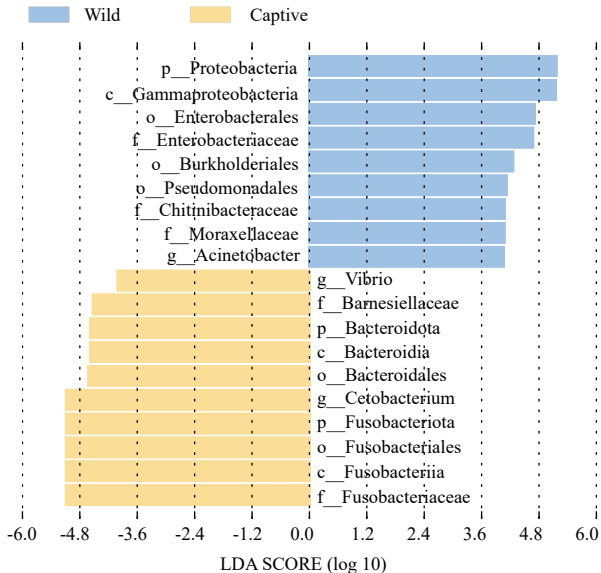

Supplement: Supplementary Figure 6 — LDA Effect Size (LEfSe) method to find out microbiota biomarkers in wild and captive cavefish. [file Data_Sheet_6.PDF]

A

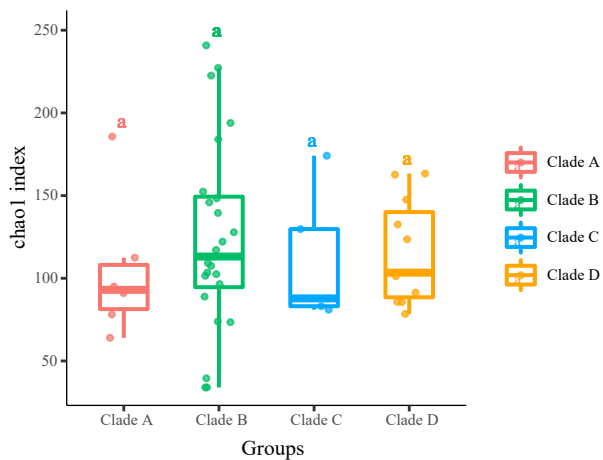

B

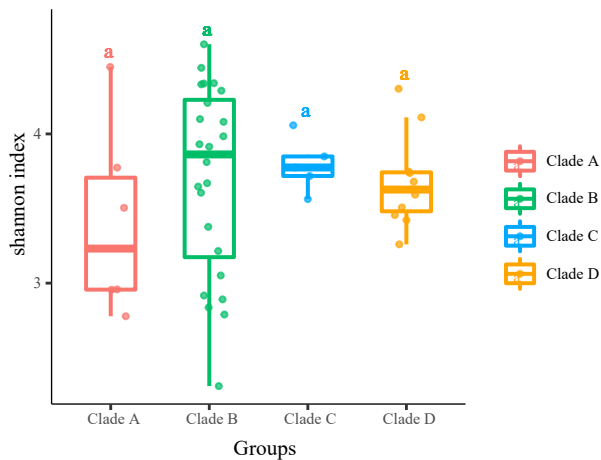

C

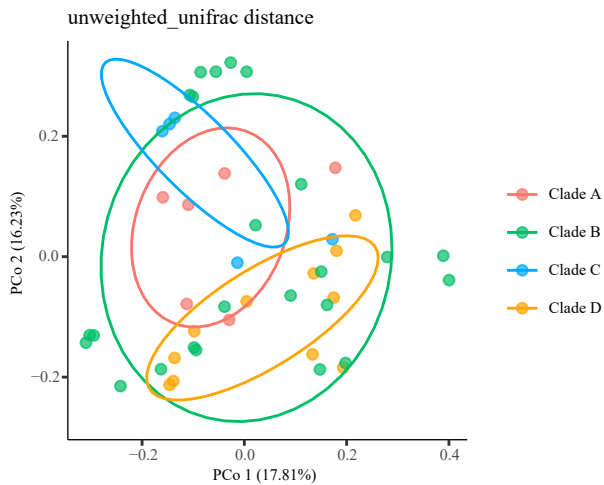

D

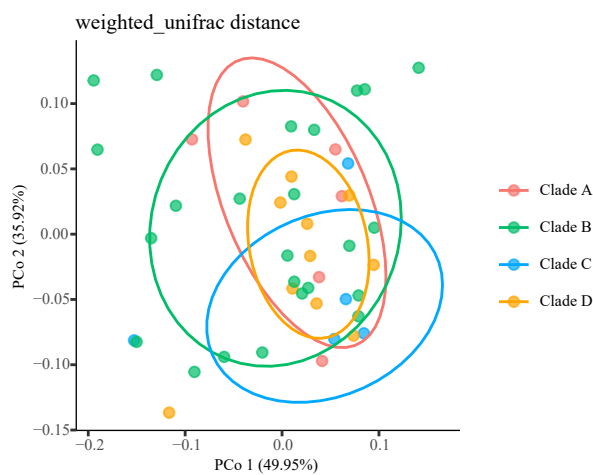

Supplement: Supplementary Figure 7 — The biodiversity of gut microbiota in species from the four phylogenetic clades after captivity. [file Data_Sheet_7.PDF]

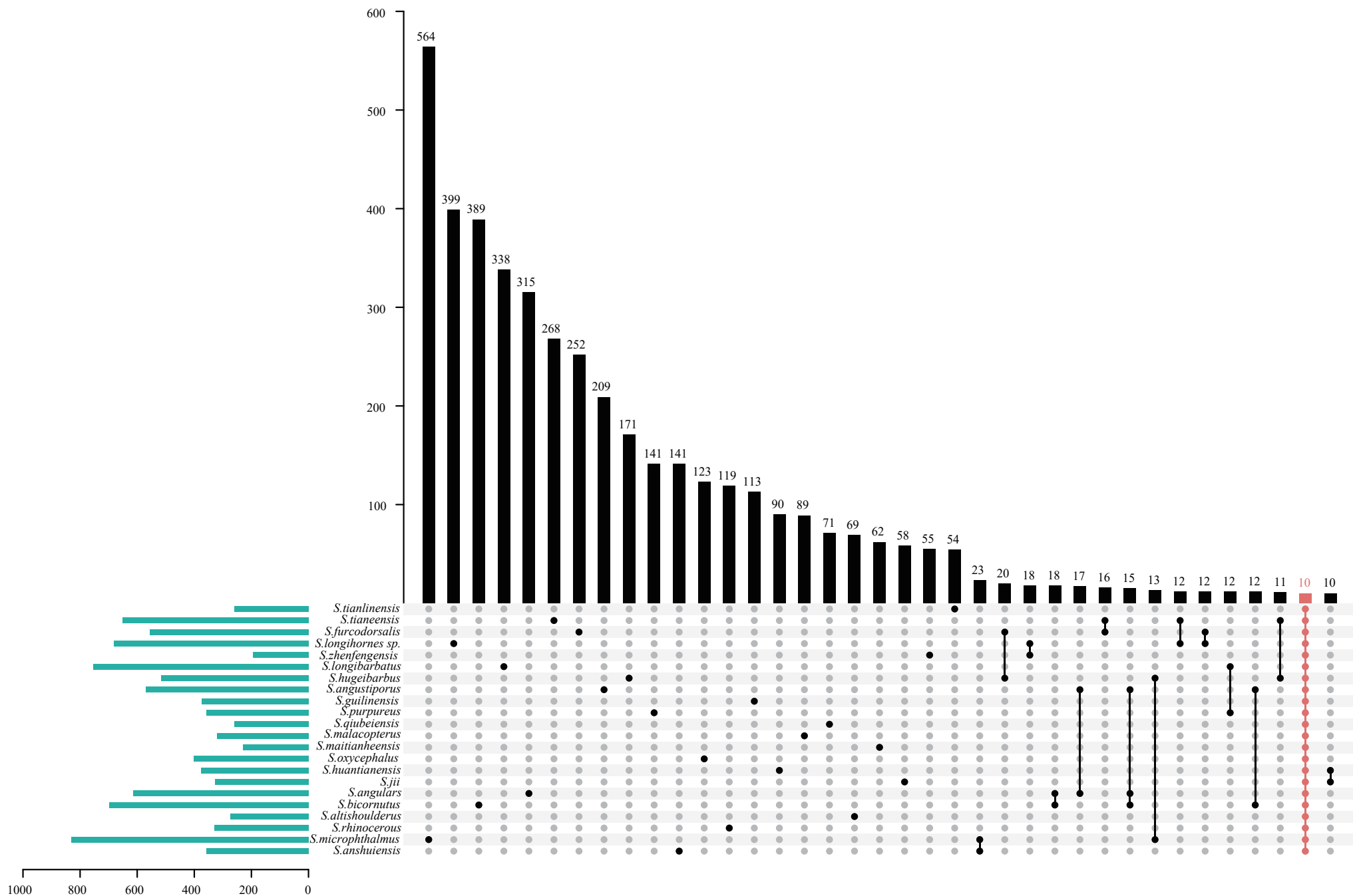

Supplement: Supplementary Figure 8 — Ten shared ASVs among 22 species of Sinocyclocheilus. [file Data_Sheet_8.PDF]

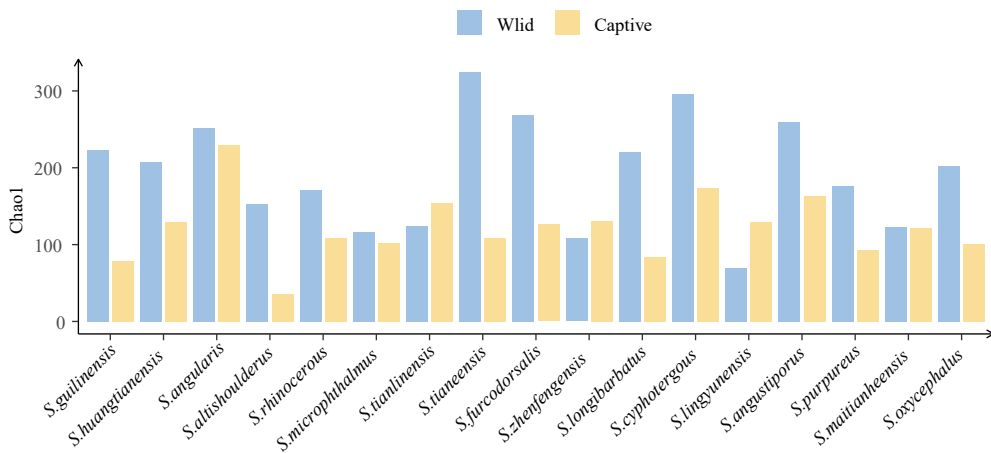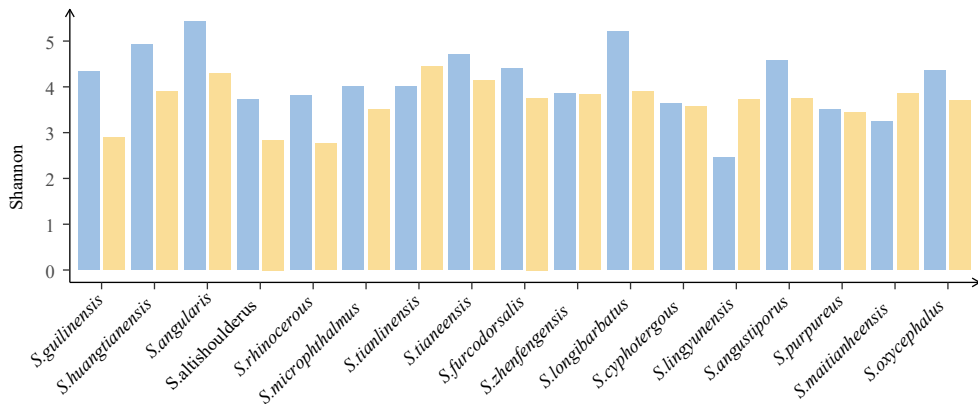

Supplement: Supplementary Figure 9 — Comparison of alpha diversity indices of Sinocyclocheilus (N = 17) before and after captivity. [file Data_Sheet_9.PDF]
